# GNG12 as A Novel Molecular Marker for the Diagnosis and Treatment of Glioma

**DOI:** 10.3389/fonc.2022.726556

**Published:** 2022-07-19

**Authors:** Runze Liu, Zhendong Liu, Yaoye Zhao, Xingbo Cheng, Binfeng Liu, Yanbiao Wang, Jialin Wang, Xiaoyu Lian, Yongjie Zhu, Yanzheng Gao

**Affiliations:** ^1^ Henan University People’s Hospital, Henan Provincial People’s Hospital, Zhengzhou, China; ^2^ Department of Surgery of Spine and Spinal Cord, Henan Provincial People’s Hospital, Henan International Joint Laboratory of Intelligentized Orthopedics Innovation and Transformation, Henan Key Laboratory for Intelligent Precision Orthopedics, People’s Hospital of Zhengzhou University, People’s Hospital of Henan University, Zhengzhou, China; ^3^ Zhengzhou University People’s Hospital, Henan Provincial People’s Hospital, Zhengzhou, China

**Keywords:** Guanine nucleotide-binding protein subunit gamma-12, prognosis, biomarker, oncogene, glioma

## Abstract

**Purpose:**

GNG12 influences a variety of tumors; however, its relationship with glioma remains unclear. The aim of this study was to comprehensively investigate the relationship between GNG12 and the clinical characteristics and prognosis of glioma patients and reveal the mechanisms causing the malignant process of GNG12.

**Materials and Methods:**

We obtained information on clinical samples from multiple databases. The expression level of GNG12 was validated using a RT-qPCR and IHC. KM curves were used to assess the correlation between the GNG12 expression and OS of glioma patients. An ROC curve was drawn to assess the predictive performance of GNG12. Univariate and multivariate Cox analyses were performed to analyze the factors affecting the prognosis of patients with glioma. GSEA and TIMER databases were used to estimate the relationship between GNG12 expression, possible molecular mechanisms, and immune cell infiltration. CMap analysis was used to screen candidate drugs for glioma. Subsequent *in vitro* experiments were used to validate the proliferation and migration of glioma cells and to explore the potential mechanisms by which GNG12 causes poor prognosis in gliomas.

**Results:**

GNG12 was overexpressed in glioma patients and GNG12 expression level correlated closely with clinical features, including age and histological type, etc. Subsequently, the K-M survival analysis indicated that the expression level of GNG12 was relevant to the prognosis of glioma, and the ROC curve implied that GNG12 can predict glioma stability. Univariate and multivariate analyses showed that GNG12 represents a risk factor for glioma occurrence. GNG12 expression is closely associated with some immune cells. Additionally, several *in vitro* experiments demonstrated that down-regulation of GNG12 expression can inhibits the proliferation and migration capacity of glioma cells. Ultimately, the results for the GSEA and WB experiments revealed that GNG12 may promote the malignant progression of gliomas by regulating the cell adhesion molecule cell signaling pathway.

**Conclusion:**

In this study, we identified GNG12 as a novel oncogene elevated in gliomas. Reducing GNG12 expression inhibits the proliferation and migration of glioma cells. In summary, GNG12 can be used as a novel biomarker for the early diagnosis of human gliomas and as a potential therapeutic target.

## Introduction

Gliomas represent the most common primary intracranial malignant tumors found in adults, accounting for 81% of intracranial brain malignancies (1). An increasing number of scholars have studied this disease because it is difficult to detect at its early onset and has a poor prognosis. According to the fifth revised edition of the Guidelines for Central Nervous System Tumors published by the World Health Organization (WHO) in 2016, gliomas are mainly divided into two categories: diffuse and non-diffuse under restricted growth patterns. With progress in research on the molecular mechanisms of glioma, glioma subtypes have gradually shifted from histological classifications, such as diffuse astrocytomas and oligodendroglial cell tumors, to molecular classifications, such as isocitrate dehydrogenase 1 and 2 (IDH1/IDH2) point mutations and 1p/19q co-deletions (2). Temozolomide is often used to administer chemotherapy within 6 months of surgery for high-grade gliomas (3, 4), and combining this treatment with radiation therapy is becoming widely accepted by clinicians and patients (5). Although patients with low-grade gliomas tend to have a better prognosis after surgical resection, some studies have shown that a better prognosis would be obtained in combination with radiotherapy or chemotherapy (4, 6). In addition, patients with IDH mutations or 1p/19q co-deletions have had mutated genes that were highly sensitive to alkylating agents, so their survival time after temozolomide chemotherapy tended to be longer than that of patients with wild-type IDH or without 1p/19q co-deletions (7). Thus, an increasing number of molecular targets are being used in clinical diagnosis and treatment, and they play a crucial role in managing various types of gliomas. Despite several treatment options being available, the 5-year survival rate of patients with diffuse glioma remains poor. Therefore, the search for increasingly effective and highly specific biomarkers is urgently needed to improve this discouraging situation.

Guanine nucleotide-binding proteins (G proteins) are a family of signaling proteins composed of α, β, and γ subunits that can bind to guanosine diphosphate and demonstrate GTP hydrolase activity, which functions as a molecular switch during signal transduction (8). GNG12 belongs to the G protein family and influences cellular functions such as cell division, differentiation, and metastasis (9, 10). Previous studies have shown that BV-2 protects neurons as an immune cell within the nervous system, and after knocking down GNG12 in BV-2 cells, the expression of the inflammation-related factor TNF-α increases (11). Therefore, GNG12 acts as a regulatory factor that inhibits inflammation. GNG12 is not only involved in the inflammatory response; in recent years, the relationship between G proteins and tumors has received considerable attention. Many studies have confirmed that some members of the G protein family strongly influence the pathology of cancer. Notably, GNG12 overexpression regulates PD-L1 expressions by activating the NF-κB signaling pathway and promoting the proliferation of pancreatic cancer cells, thus leading to a poor prognosis (12). GNG12 is involved in the malignant process of osteosarcoma (13). Therefore, GNG12 may influence the development and progression of malignant tumors, and may be used as a potential biomarker for prognostic evaluation and treatment. However, there is no evidence of the function of GNG12 in brain malignancies, especially in gliomas. Thus, it is important to further explore the role of GNG12 in gliomas.

In summary, our study analyzes a large sample of data from multiple databases. This is the first study to explore the relationship between GNG12 expression levels and the clinical features and prognosis of gliomas. Concurrently, we used basic experimental validation to reveal the role of GNG12 in the disease progression of gliomas and some of the mechanisms leading to poor prognoses. Therefore, we believe that this study will provide a new biomarker for prognostic assessments of gliomas and a new target for gene therapy to benefit patients with glioma.

## Materials and Methods

### Data Collection

The Gene Expression Profiling Interactive Analysis (GEPIA, http://gepia.cancer-pku.cn) is an interactive online analysis platform developed by Peking University. The platform contains a large amount of RNA sequencing data from human tumor tissues and mutually matched normal tissues (14). Based on this platform, we analyzed the expression levels of GNG12 in some common tumors and then used box plots to compare the differences in GNG12 expressions between tumor tissues (n=163) and normal brain tissues (n=207). The Gene Expression Omnibus (GEO, https://www.ncbi.nlm.nih.gov/geo/) contains high-throughput gene expression data submitted by research institutions worldwide and provides a range of web-based interfaces and applications (15). To explore GNG12 expression levels in gliomas, we examined microarray data from two datasets: GSE4290 (glioma=77, normal=23) and GSE50161 (glioma=34, normal=13). RNA sequencing data from 1,018 gliomas and their corresponding clinical information were obtained from the Chinese Glioma Genome Atlas (CGGA, http://www.cgga.org.cn) database. From the 1,018 glioma samples, patients with complete relevant clinical information were selected for this study (**Supplementary Table 1**) (16). Based on the Human Protein Atlas (HPA; https://www.proteinatlas.org/), an immunohistochemical database, the differential expression of GNG12 between glioma and control groups was explored at the protein level (17). The IVY-GAP database (http://glioblastoma.alleninstitute.org/), an immunofluorescence database, was used to explore the differential expression of GNG12 between the glioma and control groups at the nucleic acid level.

### Patients and Tissue Preparation

From June 2019 to September 2019, tissue samples were collected from 24 patients with glioma and seven patients with epilepsy at Henan Provincial People’s Hospital (Zhengzhou, China). All the samples contained complete clinical information about the patients. Samples were obtained surgically by dividing the tissue into 1 cubic centimeter sizes, and then placing them in liquid nitrogen for freezing and storage at -80°C until total RNA was isolated. Real-time quantitative polymerase chain reaction (RT-qPCR) was used to verify the expression levels of GNG12 in glioma and non-tumor brain tissues. The study protocol was approved by the Ethics Committee of Henan Provincial People’s Hospital (Zhengzhou, China). All experiments were performed according to the guidelines approved by Henan Provincial People’s Hospital.

### Cell Culture and Transfection

Human glioma cell (LN229) were purchased from Wuhan Procell Biotechnology (Wuhan, China). Cells were incubated at 37°C in an incubator with a gas environment of 95% O2 and 5% CO2, and the medium used was Dulbecco’s modified eagle medium (DMEM) (Procell, Wuhan, China) containing 10% fetal bovine serum (Gibco, USA) and a 1% penicillin-streptomycin mixture (Procell Wuhan, China); next, the cells were passaged and reserved. Transient transfection was used in this study, and the transfection reagent was Lipo3000 (Thermo Fisher, USA). Cells were evenly inoculated into 6-well plates, and then a mixture of siRNA-Mate (GenePharma, Shanghai, China) and lipo3000 was added to each well separately. Transfection was performed using serum-free medium, and the complete medium was replaced after 6 h of action. The knockdown efficiency was determined *via* RT-qPCR after the siRNA was functional (approximately 36 h). The dishes with siRNA-NC were added as a blank control (NC), and those with siRNA-1, siRNA-2, and siRNA-3 were added as the experimental group (KD). Sequences with the highest siRNA knockdown efficiency were selected for subsequent experiments (primers and siRNA sequences are listed in **Table S2**).

### RNA Extraction and RT-qPCR

After transfection with Si-RNA, total RNA was isolated from sample tissues and corresponding cell lines using TRIzol^®^ (Invitrogen, Thermo Fisher Scientific, USA). Then, the RNA’s concentration was determined using a NanoDrop One spectrophotometer (Thermo Fisher Scientific, USA), and reverse transcription was performed to obtain cDNA (Novoprotein). Finally, the expression level of GNG12 was determined *via* RT-qPCR using the NovoStart SYBR qPCR SuperMix Plus (Novoprotein). Primers for GAPDH and GNG12 were purchased from Henan Shangya Biotechnology Co. Ltd., with the following sequences: GAPDH-F:5’-CAAGGTCATCCATGACAACTTTG-3’, GAPDH-R:5’-GTCCACCACCCTGTTGCTGTAG-3’, GNG12-F:5’-GAGCCCTTAGAGACCGAG -3’, GNG12-R:5’-AGACTTTGTGTGGTCCAATGT-3’. The thermal cycling conditions were as follows: initial denaturation at 95°C for 10 min, denaturation at 95°C for 10 s, and annealing and extension at 60°C for 30 s for a total of 40 cycles.

### Cell Counting Kit‐8 (CCK-8) Assay

Untreated LN229 cells were inoculated in 96-well plates (1000 cells/well), and after waiting for wall attachment and interference with siRNA, the absorbances at 0, 24, 48, 72, and 96 h after transfection were measured. The absorbance at 450 nm was measured using an enzyme marker after incubation in a 37°C incubator for 4 h prior to each measurement.

### Immunochemical Staining

For immunohistochemical staining (IHC), paraffin sections with a thickness of 4 μm were first dewaxed by placing them in an oven at 55°C for 1 h and then in xylene and concentration gradient ethanol for dewaxing and hydration. Antigen repair was performed in an ethylenediaminetetraacetic acid (EDTA) buffer (pH 8.0) with microwave heating for 18 min. Blocking was then performed using 10% goat serum to reduce nonspecific staining. GNG12 (1:100, Bioss, Beijing, China) primary antibody working solution was added dropwise to the slides and placed in a wet box overnight at 4°C. The following day, the secondary antibody was washed with PBST and incubated for 1 h. The exposed GNG12 protein was then labeled with 0.01% DAB chromogenic solution and the nuclei were stained with hematoxylin. Finally, the staining results were observed under a light microscope at 400x magnification, and five fields of view were selected for photography. The IHC results were processed using ImageProPlus (version 6.0). To verify the effect of GNG12 on cell proliferation, we performed cellular immunofluorescence analysis using Ki67. An equal number of LN229 glioma cells were first inoculated uniformly in 3 cm culture dishes, and the culture medium was discarded after 36 h of transfection, fixed with 4% paraformaldehyde for 10 min at room temperature, and then permeabilized with 0.5% Triton X-100 for 30 min. After washing thrice with PBS, the Ki-67 primary antibody (1:200, Abcam, China) was incubated at 4°C for 24 h. Following this incubation, the cells were washed thrice with PBS for 5 min each, and then incubated with the DyLight 594Ig G (1:200, Invitrogen, USA) secondary antibody at room temperature and protected from light for 1 h. Subsequently, the nuclei were stained with DAPI for 10 min. Finally, the Ki67 expression levels in the experimental and control groups were observed by fluorescence microscopy and photographed.

### Scratch Wound Healing Assay

Experiments were performed to verify whether GNG had any effect on glioma cell migration. Equal amounts of cells were uniformly inoculated into 6-well plates. The experimental and control groups were set, and three wells were used for parallel experiments. After the cells reached 70% confluency, the experimental group was transfected with siRNA-GNG12, and the control group was transfected with siRNA-NC. Once the cells in the culture dish were fully grown, three parallel vertical lines were drawn in each of the six wells with a 200 μL sterile spiking gun tip, and the detached cells were washed with PBS. A 2 mL sample of serum-free medium was added to each well; next, a 0 h sample was taken with an inverted microscope at 200x magnification as the first experimental data, and then placed in a 37°C incubator for 48 h. Scratches were then taken at the same position as the previous one.

### Gene Set Enrichment Analysis (GSEA)

Gene Set Enrichment Analysis(GSEA) is an ideal bioinformatics analysis tool developed by the research team of MIT and Harvard University’s Broad Institute and is used to analyze cell signaling pathways. The RNA-sequencing data obtained from the CGGA database were batch corrected and normalized using SVA and LIMMA, and then divided into the “H” (high expression) or “L” (low expression) groups according to GNG12 expression levels. The GSEA (v.4.0.3) software was used for enrichment analysis; the number of permutations was set to 1000, and the “KEGG cell signaling pathway” was selected as the gene set database.

### Western Blotting

The transfected cells were added to RIPA lysate and protease inhibitor to extract total protein (EpiZyme, Shanghai, China). After lysis on ice for 30 min, the proteins were centrifuged at 12000 rpm for 15 min at 4°C. The loading volume of each sample was measured using a BCA kit (GenStar, Beijing, China). Identical masses of proteins were separated using sodium dodecyl sulfate-polyacrylamide gel electrophoresis (SDS-PAGE). Subsequently, the proteins were transferred to PVDF membranes (Bio-Rad, UK) and sealed with skim milk powder. PVDF membranes were incubated overnight at 4°C with primary antibodies against VCAM-1 (1:500, Proteintech, USA), ICAM-1 (1:2500, Proteintech, USA), CDH2 (1:1500, Proteintech, USA), and GAPDH (1:10000, Proteintech, USA). Finally, the HRP-labeled goat anti-mouse secondary antibody (1:5000, Proteintech, USA) was incubated with the filter membrane for 1 h. GNG12 protein expression levels were detected using an imager and a chemiluminescent substrate (ECL) kit (Thermo Fisher Scientific, USA).

### Co-Expression and Drug Analysis

Gene co-expression is an analytical method that uses a large amount of gene expression data to construct correlations between genes and gene functions. The co-expression analysis of GNG12 was performed using Pearson’s method. According to the correlation coefficients, the P-value obtained ten genes were positively and negatively correlated with GNG12. Based on the positive and negative related top 10 genes, the complex algorithm of CMap (Connectivity Map, https://portals.broadinstitute.org/cmap) was used to obtain related genes that may downregulate the expression level of GNG12 and screen GNG12 gene therapy drugs in the PubChem database (https://pubchem.ncbi.nlm.nih.gov); this information included the name and chemical formula of the drug and 2D and 3D structures.

### TIMER Database Analysis

The Tumor Immune Estimation Resource (TIMER, https://cistrome.shinyapps.io/timer) is a rich database of tumor immunology and genetics, including gene expression, mutation, and copy number variation (18). In this study, we evaluated the association of GNG12 expression with the infiltration of six different immune cell types (B cells, CD4 + T cells, CD8 + T cells, macrophages, neutrophils, and dendritic cells).

### Statistical Analysis

R software (v.3.6.1) was used to perform the statistical data analysis. Survival and clinical characteristic data were obtained from the CGGA database. The overall survival of GNG12 was determined *via* COX regression and the Kaplan-Meier(KM) method. In addition, Wilcox or Kruskal tests were used to test the relationship between the expression level of GNG12 and clinical characteristics. A time-dependent receiver operating characteristic (ROC) curve shows the evaluation value of the clinical prognosis of GNG12 for glioma. Univariate and multivariate analyses were used to analyze the factors affecting the prognosis of glioma patients. The expression differences between the experimental and control groups of GNG12 were tested using GraphPad Prism software (9.1.0) with a Mann-Whitney test, Chi-square test, or Fisher’s exact test (P<0.05, considered statistically significant).

## Results

### GNG12: Highly Expressed in Glioma

**Figure 1:**
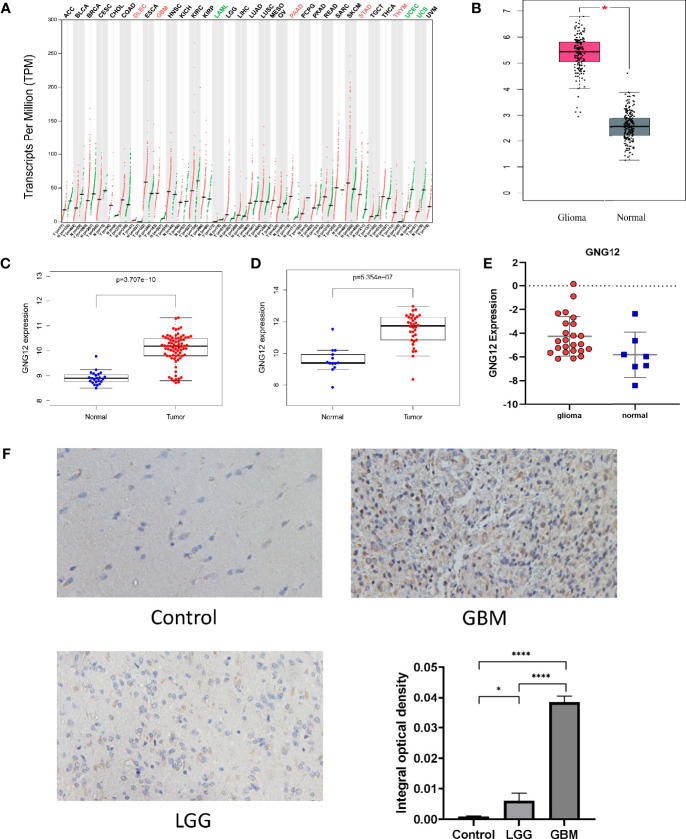
The expression of GNG12 at different levels (mRNA, protein, gene microarray and gene sequencing) in gliomas. **(A)** The expression of GNG12 in different types of tumor tissues in the GEPIA, the expression of GNG12 in glioma (n=163) and normal brain tissue (n=207), red and green respectively represent the difference in expression level. Green means that the gene is under-expressed in tumor tissues, and the red means the gene is highly expressed in tumor tissues. **(B)** In the GEPIA database, the expression of GNG12 is different in glioma and normal brain tissue. **(C)** Box plot based on the expression level of GNG12 in the GSE4290 (Glioma=77, Normal=23). **(D)** Box plot based on the expression level of GNG12 in the GSE50161(Glioma=34, Normal=13). **(E)** RT-qPCR experimental results show that the expression of GNG12 in gliomas is higher than that in normal tissues. **(F)** Results of immunohistochemical experiments in normal brain tissue, low-grade glioma and glioblastoma and statistical analysis (Magnification: *400). (****P<0.0001, *P<0.05) .

GEPIA was used to analyze the expression of GNG12 in different tumors. GNG12 was highly expressed in various tumors, including glioblastoma multiforme (GBM), lymphoid neoplasm diffuse large B-cell lymphoma (DLBC), pancreatic adenocarcinoma (PAAD), and thymoma (THYM) (**Figure 1A, B**). The GSE4290 and GSE50161 datasets from the GEO database revealed that the expression level of GNG12 was higher in gliomas than in normal brain tissue (**Figures 1C, D**). Moreover, GNG12 protein expression levels were similarly elevated in HPA immunohistochemistry and IVY-GAP *in situ* hybridization data (**Figures S1 **and** S2**, respectively). More importantly, we obtained the same results as those predicted by the database through further RT-qPCR and IHC experimental validations (**Figures 1E** **, F**). Combined with the above database analysis and experimental results, GNG12 was overexpressed in glioma.

### Clinical Characteristics of Studied Patients and Their Relationship With GNG12 Expression

**Figure 2:**
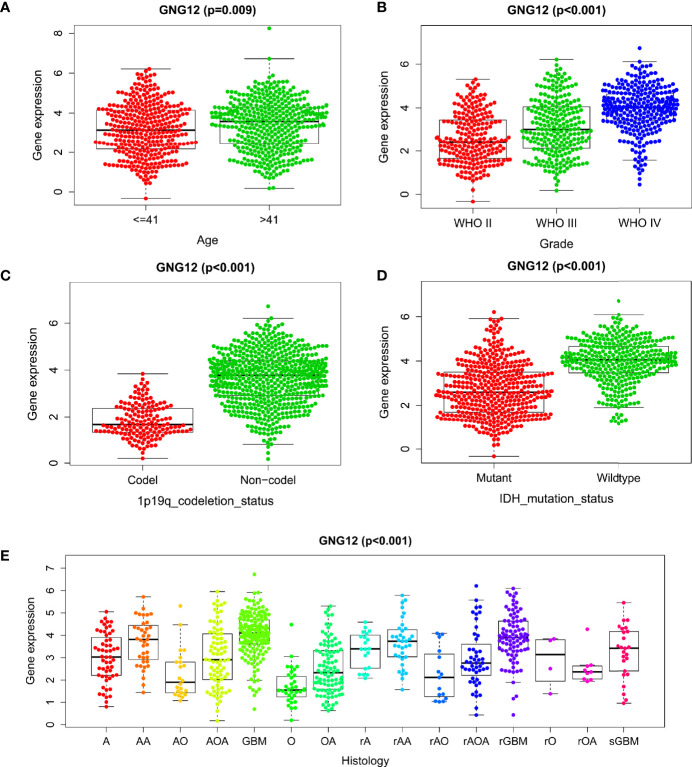
Correlation between GNG12 and different clinical characteristics based on CGGA database. **(A)** age. **(B)** WHO grade. **(C)** 1p19q co-deletion. **(D)** IDH mutation. **(E)** Histology.

Subsequently, the relationship between GNG12 expression levels and patients clinical characteristics with glioma was analyzed. Wilcoxon and Kruskal-Wallis tests were used to analyze the relationship between clinically relevant patient information and the expression levels of GNG12. We observed that the expression level of GNG12 significantly correlated with age, WHO classification, histological type, primary recurrence classification, 1p19q coding data, and IDH mutation status. As shown in **Figure 2**, the expression of GNG12 in glioma tissues from patients aged >41 years was significantly higher than that in patients aged ≤ 41 years (p =0.009). The expression level of GNG12 was positively correlated with the WHO grade of glioma (P < 0.001). Regarding the 1p19q co-deletion status, there were lower gene expression levels in patients with a codeletion of 1p19q than in patients with non-codeletion of 1p19q (p < 0.001). Moreover, for the IDH mutation status, there was a higher level of gene expression in the wild type than in the mutant (p < 0.001). In addition, recurrent gliomas had higher GNG12 expression levels than primary gliomas, which explains why patients with recurrent gliomas had a worse prognosis (p<0.05). The strong association between GNG12 expression levels and the clinical characteristics of glioma patients suggests that GNG12 may be related to the survival prognosis of glioma.

### GNG12 Associated With Poor Prognoses in Patients With Glioma

**Figure 3:**
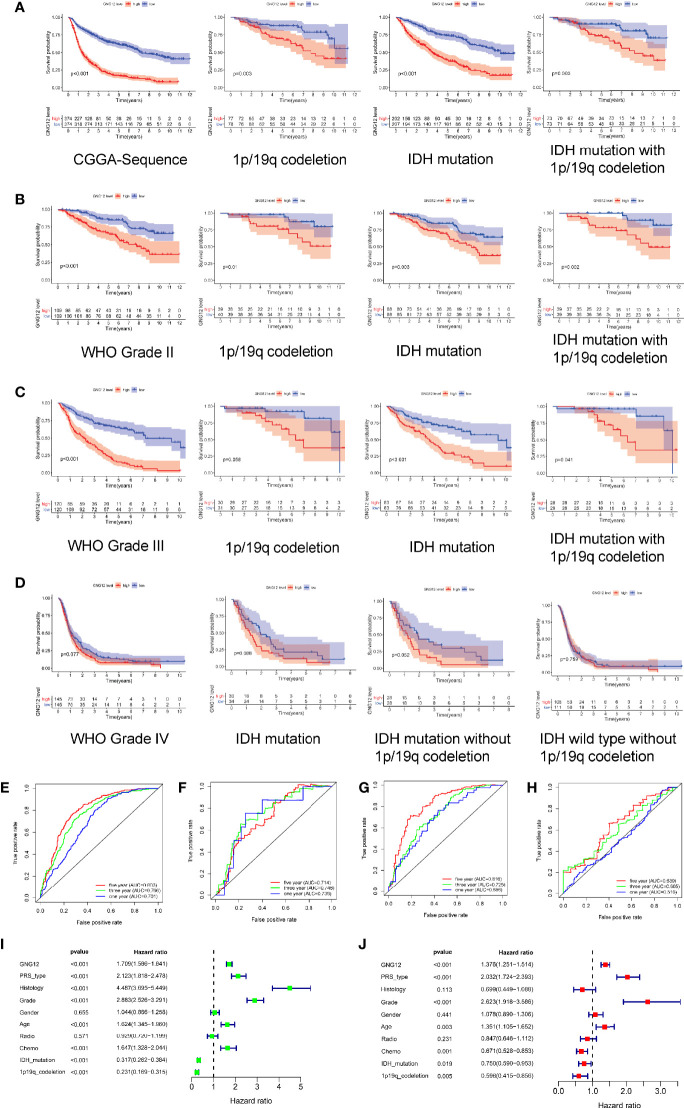
Relationship between different expression status of GNG12 and prognosis of glioma patients based on CGGA RNA-seq data. **(A)** In order, overall survival based on CGGA database, survival with 1p/19q co-deletion, survival with IDH mutation and survival with IDH mutation with 1p/19q co-deletion. **(B)** Survival rates for WHO grade 2, survival with 1p/19q co-deletion in grade 2, survival with IDH mutation and survival with IDH mutation with 1p/19q co-deletion, in that order. **(C)** In order, the survival rate of WHO grade 3, the survival rate of 1p/19q co-deletion in grade 3, the survival rate of IDH mutation and the survival rate of IDH mutation with 1p/19q co-deletion. **(D)** In order, the survival rate of WHO grade 4, the survival rate of IDH mutation in grade 4, the survival rate of IDH mutation without 1p/19q co-deletion and the survival rate of IDH wild type without 1p/19q co-deletion. **(E–H)** ROC curve shows that GNG12 has good diagnostic value in glioma, ROC curves of overall glioma based on CGGA and different WHO classifications. **(I, J)** Analysis of univariate and multivariate factors affecting the prognosis of patients with glioma. **(I)** Univariate regression analysis; **(J)** Multivariate analysis.

We further explored the relationship between GNG12 expression levels and the survival prognoses of patients with glioma. First, the survival analysis results showed a significant correlation between high GNG12 expression and reduced survival rates of patients with glioma. Subsequently, the survival rates of the different molecular subtypes in each grade were analyzed separately. In grade II and III gliomas and in all samples, the results showed that the survival rate of the GNG12 high expression group was lower than that of the low expression group, regardless of the presence of IDH mutations or 1p/19q co-deletions (**Figures 3A** **–C**). In grade IV gliomas, the results were not statistically significant, which probably resulted from the poor prognoses of high-grade gliomas and the associated lower survival rate of the patients (**Figure 3D**). However, we nonetheless observed an overall trend that was consistent with the results of the analysis of grade II and III gliomas. Moreover, ROC analysis showed that the area under the ROC curve (AUC) was 0.701, 0.766, and 0.803 for the one, three, and five-year Overall Survival (OS), respectively (**Figure 3E**). The AUC data were meaningful for different WHO classifications (**Figures 3F** **–H**). Therefore, our results indicate that GNG12 may serve as a biomarker for glioma, especially in the five-year OS group. Univariate and multivariate analyses showed that high levels of GNG12 expression, PRS grading (p < 0.001; HR = 2.032; 95% CI, 1.724-2.393), WHO grading (p < 0.001; HR = 2. 623; 95% CI, 1.918-3.586) and age (p < 0.003; HR = 1.351. 95% CI, 1.105-1.652) may represent an independent risk factor for poor prognoses in patients with glioma. IDH mutation status (p < 0.019; HR = 0.750; 95% CI, 0.590-0.953) and 1p/19q co-deletion (p < 0.005; HR = 0.596; 95% CI, 0.415-0.856) may represent protective factors (**Figures 3I, J**, respectively). These data indicate that GNG12 may serve as a predictive biomarker for poor prognosis.

### Correlations of GNG12 With Immune Cell Infiltration

**Figure 4:**
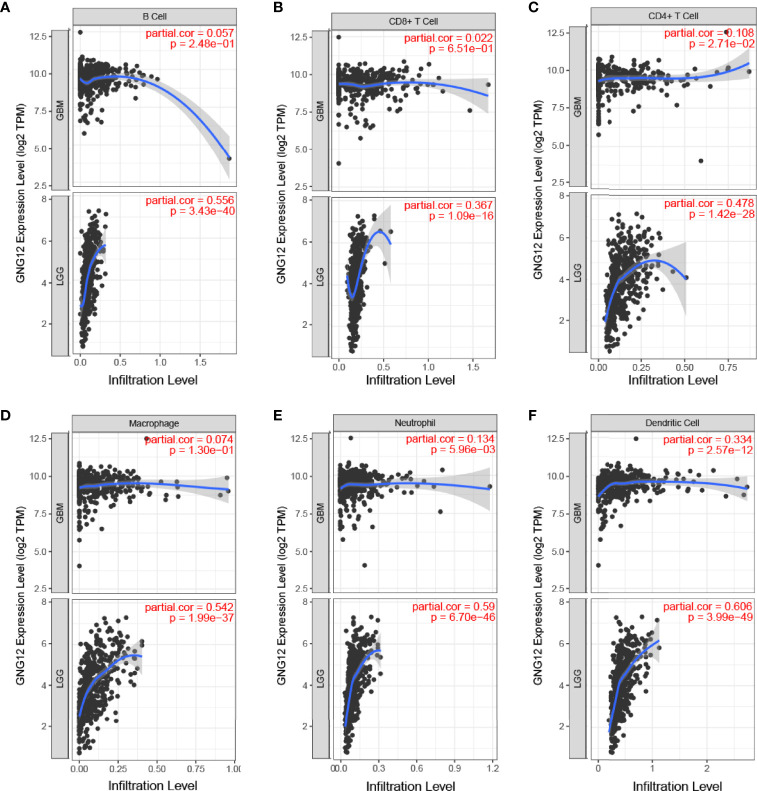
Relationship between expression of the GNG12 gene and proportion of immune infiltrates. **(A)** B Cell **(B)** CD8+T Cell **(C)** CD4+T Cell **(D) **Macrophage **(E)** Neutrophil **(F)** Dendritic Cell.

The correlation between GNG12 expression levels and tumor immune cell infiltration was analyzed using the TIMER database. After adjusting for purity, the expression levels of GNG12 in GBM and LGG correlated positively with the degree of infiltration of CD4^+^ T cells (P = 2.71e-02, GBM; P = 1.42e-28, LGG), dendritic cells (P = 2.57e-12, GBM; P = 3.99e-49, LGG), and neutrophils (P = 5.96e-03, GBM; P = 6.70 e-46, LGG) (**Figure 4**). In addition, GNG12 expression was associated with the degree of infiltration of B cells (P = 3.43e-40, LGG), CD8^+^ T cells (P = 1.09e-16, LGG), and macrophages (P = 1.99e-37, LGG), but no significant differences were found in GBM. In glioma patients, a loss of the GNG12 copy number resulted in decreased infiltration of CD4^+^ T cells, macrophages, neutrophils, and dendritic cells (**Figure S5**). In addition, the infiltration of B cells and CD8^+^ T cells also decreased in the LGG. Therefore, we further used Kaplan-Meier curves to verify our hypothesis (**Figure S4**), and the results indicated that high GNG12 expression level in enriched B cells, CD8^+^ T cells, CD4^+^ T cells, macrophages, neutrophils, and dendritic cells were associated with worse prognoses in patients with LGG, whereas high GNG12 expression level in enriched dendritic cells were associated with poor overall survival outcomes. In addition, owing to the broad prospects of immunotherapy, we further determined the relationship between the GNG12 expression and PD-1, PD-L1, and PD-L2 expressions. Encouragingly, GNG12 was positively correlated with PD-L1 (r = 0.293, P = 5.23e-04) and PD-L2 (r = 0.22, P = 9.73e-03) in GBM. We also found that GNG12 was positively correlated with PD-1 (r = 0.347, P = 5.76e-15), PD-L1 (r = 0.474, P = 4.39e-28), and PD-L2 (r = 0.684, P = 4.01e-67) in LGG (**Figure S4**). In conclusion, it is possible that a high GNG12 expression leads to reduced immune cell infiltration and may be an important factor contributing to poor prognoses in patients with glioma.

### Co-Expression Analysis and Medical Therapy Related to GNG12

**Figure 5:**
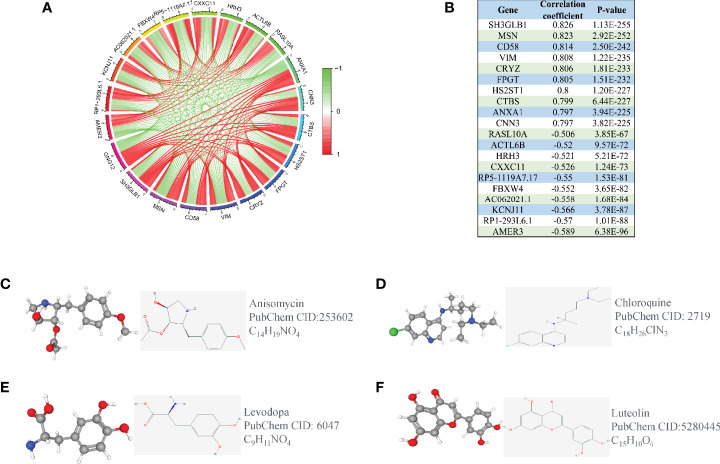
Co-expression analysis of GNG12. **(A)** The ten most significant genes of positive and negative correlating with GNG12; **(B)** The correlation coefficients and P values of the ten most important positive and negative genes related to GNG12. Screening of gene therapy drugs for GNG12 in the CMap and PubChem database (Drug name, chemical structure, 2D structure, 3D structures). **(C)** Anisomycin **(D)** Chloroquine **(E)** Levodopa **(F)** Luteolin.

To explore the related genes that could have positive or negative regulatory effects on GNG12, we performed a co-expression analysis. The genes that positively regulated GNG12 were SH3GLB1, MSN, CD58, VIM, CRYZ, FPGT, HS2ST1, CTBS, ANXA1, and CNN3. The negatively regulated genes were RASL10A, ACTL6B, HRH3, CXXC11, RP5-1119A7.17, FBXW4, AC062021.1, KCNJ11, RP1-293L6.1, and AMER3 (**Figures 5A** **, B**). We also screened for gene therapy drugs for GNG12 in the CMap and PubChem databases. Four possible gene therapy drugs were identified for GNG12: anisomycin, chloroquine, levodopa, luteolin (**Figures 5C** **–F**).

### Knockdown of GNG12 Expression Level Inhibits the Proliferation and Migration of Glioma Cells

**Figure 6:**
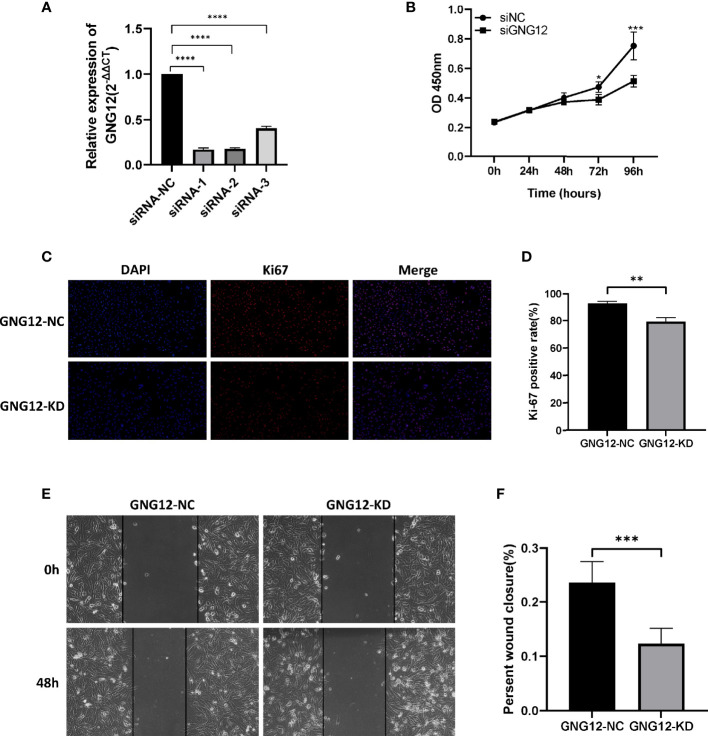
Effects on proliferation and migration of glioma cells by down-regulating GNG12 expression levels. **(A)** Detection of knockdown efficiency of different siRNA sequences in the glioma cell line LN229. **(B)** CCK-8 assay to compare the effect on cell proliferation after down-regulation of GNG12. **(C)** Immunofluorescence assay comparing the positive rate of Ki-67 after transfection with siRNA-NC and siRNA-KD (Magnification: *400). **(D)** Immunofluorescence assay to analyze the results. **(E)** Cell scratch healing assay using LN229 cell line to compare the migration distance between GNG12-NC group and GNG12-KD group (Magnification: *200). **(F)** Results of statistical analysis of cell scratching experiments. (****P<0.0001, ***P<0.001, **P<0.01, *P<0.05).

To further validate the effect of GNG12 on glioma, we performed a series of *in vitro* experiments. First, we designed three small molecule-interfering RNAs to inhibit the expression of GNG12 in glioma cells. The results showed that siRNA-1 was screened with the highest knockdown efficiency using RT-qPCR; therefore, we selected siRNA-1 for the subsequent target downregulation of GNG12 (**Figure 6A**). Knocking down the expression level of GNG12 clearly affected the proliferation and migration abilities of glioma cells. The results of the CCK-8 assay showed that the proliferation efficiency of glioma cells in the KD group was significantly lower than that in the NC group (**Figure 6B**). In parallel, the Ki-67 immunofluorescence assay suggested that the Ki-67 expression level was higher in the NC group than in the KD group, and the differences between the groups were statistically significant (**Figures 6C, D**, respectively). The diminished migratory capacity of glioma cells within 48 h of GNG12 downregulation was ultimately verified using a cell scratch healing assay (**Figures 6E** **, F**). In summary, our targeted downregulation of GNG12 expression levels in glioma cell lines resulted in a certain degree of diminished proliferation and migration; therefore, we speculate that a high GNG12 expression may be an important factor in the poor prognoses of glioma patients.

### Regulation of Cell Adhesion Molecules Cell Signaling Pathway Proteins by GNG12

**Figure 7:**
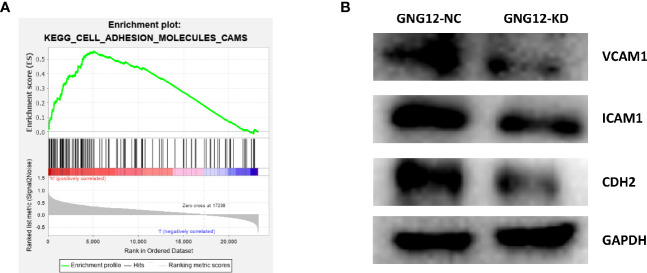
The involvement of GNG12 in the regulation of cell adhesion molecule signaling pathway. **(A)** Analysis of the significant enrichment of GNG12 in cell adhesion molecule signaling pathway based on GSEA. **(B)** Western blotting assay to detect the effect of knockdown of GNG12 on the expression of VCAM-1, ICAM-1, CDH2.

We performed a GSEA to further explore the potential mechanisms through which GNG12 affects the malignant biological behavior of glioma cells. The results showed that GNG12 was enriched in tumor-related pathways, including the cell adhesion molecule signaling pathway, JAK-STAT signaling pathway (**Figure 7A**), TOLL-LIKE receptor signaling pathway, focal adhesion, VEGF signaling pathway, and MAPK signaling pathway (**Figure S6**). These cellular signaling pathways showed significant different enrichment rates in samples from patients showing the GNG12 high-expression phenotype based on NES, NOM P-values, and FDR values (**Table 1**), thus indicating a potential role for GNG12 in developing glioma. To probe the specific mechanism by which GNG12 leads to poor prognosis of glioma, we verified the predicted results of GSEA in WB experiments. After the GNG12 expression level was knocked down and the NC group was used for comparison, the assay revealed that the protein expression levels of VCAM-1 and CDH2 in the cell adhesion molecule pathway were significantly decreased in the KD group, and the ICAM-1 protein expression level was also decreased to an extent (**Figure 7B**). This suggests that GNG12 may help regulate the cell adhesion molecule pathway involved in the malignant process of gliomas.

**Table 1:** The gene set enriches the high GNG12 expression phenotype.

Gene set name	NES	NOM p-val	FDR q-val
KEGG JAK STAT SIGNALING PATHWAY	1.86	0.006	0.050
KEGG TOLL LIKE RECEPTOR SIGNALING PATHWAY	1.89	0.006	0.042
KEGG FOCAL ADHESION	1.95	0.004	0.051
KEGG VEGF SIGNALING PATHWAY	1.76	0.004	0.070
KEGG MAPK SIGNALING PATHWAY	1.67	0.012	0.078
KEGG CELL ADHESION MOLECULES CAMS	1.83	0.0041	0.058

Gene sets with NOM P-value <0.05 and FDR q-value <0.25 were considered as significantly enriched.

NES, normalized enrichment score; NOM, nominal; FDR, false discovery rate; GNG12, Guanine nucleotide-binding protein subunit gamma-12.

## Discussion

Diffuse glioma cells are characterized by extreme invasiveness, which leads to poor prognoses in patients with glioma (19). Therefore, there is a need to identify biomarkers with a high sensitivity and. Most recent studies have shown that GNG12, as a novel biomarker, plays a key regulatory role in the malignant behavior of tumors. For example, GNG12 can promote pancreatic cancer cell growth *in vitro* and *in vivo* by activating the NF-βB/PD-L1 signaling axis (12). However, the relationship between GNG12 and glioma prognoses as well as related clinical features has not received much attention. Therefore, this study utilized a multi-omics approach and comprehensive bioinformatics analysis to explore the relationship between GNG12 and the malignant biological behavior of gliomas.

To explore this relationship, we performed various bioinformatics analyses. First, using the GEPIA, GEO, and HPA databases, we found that the expression level of GNG12 was significantly increased in gliomas. Our RT-qPCR and IHC results further verified that GNG12 was consistently overexpressed in gliomas. Second, this study showed that high expression r level of GNG12 were significantly correlated with related clinical features, such as age, WHO classification, and molecular typing (20). Interestingly, the results of some studies are similar to ours. For example, Juan Li confirmed that GNG12, as an oncogene in pancreatic cancer and lung cancer, was closely associated with clinical features, and predicted poor prognoses in patients (12, 21). Survival analysis showed that overexpressing GNG12 reduced overall survival rates in patients with grade II and III gliomas, and the same conclusion was obtained for more detailed typing, such as IDH mutation status and 1p/19q co-deletion status; this is a finding that has diagnostic value for prognoses using an ROC curve. However, there was no significant difference in GNG12 expression level among the grade IV gliomas. A possible explanation for this phenomenon is that GBM, one of the most malignant tumors in the human body, is influenced by several genetic and environmental factors (2). Finally, we excluded the influence of random factors *via* univariate and multivariate analyses, from which we reached the scientific conclusion that a high GNG12 expression level may serve as an important predictor of poor prognoses in patients with glioma. Thus, based on the above studies, GNG12 may represent a risk factor for poor glioma prognoses; however, its pathological mechanism needs to be explored in depth.

Previous studies have shown that tumor-infiltrating immune cells are important entities in the tumor microenvironment and are closely associated with the malignant biological behavior of gliomas and patient survival rates (22–25). To explore the relationship between GNG12 and immune cell infiltration, we analyzed the correlation between the expression level of GNG12 and the infiltration level of glioma immune cells using TIMER. The results showed that GNG12 is associated with the infiltration of various immune cells in gliomas, especially B cells, CD4^+^ T cells, macrophages, and dendritic cells in low-grade gliomas. Although there have been no studies evaluating the correlation between GNG12 and immune cells, the correlation between single genes and cancer immunity has been extensively studied. For example, there is a positive correlation between JAK1 and the infiltration of immune cells, such as CD8^+^ T cells and dendritic cells in breast cancer (26). CD70 promotes macrophage infiltration into glioma (27). Interestingly, our study’s GNG12 expression was positively correlated with immune checkpoints, including PD-1, PDL-1, and PD-L2 in gliomas; therefore, we can combine this gene with immune checkpoint inhibitors to provide a novel glioma immunotherapy. These combined results suggest that GNG12 may interfere with the tumor microenvironment of gliomas by affecting the infiltration of various immune cells, which in turn leads to the development and poor prognosis of gliomas.

To explore small-molecule drugs that could potentially inhibit GNG12, this study screened four small-molecule compounds with potential therapeutic effects on glioma using a CMap analysis. Each drug was obtained through the PubChem database and showed varying degrees of antitumor properties. For example, anisomycin, a monohydroxypyrrolidine and organonitrogen heterocyclic antibiotic, interferes with protein and DNA synthesis by inhibiting the peptidyl transferase or 80S ribosomal system. Previous studies have confirmed that anisomycin promotes cell apoptosis through regulating PP2A/C secretion and plays an important role in the treatment of glioma (28). Some researchers have also found that anisomycin may enhance tumor necrosis factor-related apoptosis-inducing ligand (TRAIL)-induced apoptosis in kidney cancer cells by downregulating the expressions of Bcl-2, c-FLIP(L), and Mcl-1 (29). This directly or indirectly demonstrates the active anticancer properties of anisomycin in different types of tumors and suggests its promise for eventual clinical applications. In addition, lignocaine has demonstrated antioxidant, antitumor, and immunomodulatory effects in treatments of tumors and may act as an angiogenesis inhibitor through exerting anti-tumor effects. However, lignocaine is poorly hydrophilic; therefore, researchers have combined it with folic acid-modified polyethylene glycol PCL nanoparticles for application. Surprisingly, a significant inhibition of glioma angiogenesis and tumor cell proliferation was observed (30). The reliability of the CMap tool for drug predictions has therefore been demonstrated by previous studies (31). This approach expands the indications for drugs that have not yet been investigated in tumors, and may provide a new direction for subsequent glioma drug therapy.

We conducted a series of experiments to explore the effect of GNG12 on gliomas. The *in vitro* results showed that downregulating GNG12 expression level inhibited the proliferation and migration ability of gliomas, which may represent a potential link between poor glioma prognoses and GNG12. To further understand the pathological mechanism by which GNG12 causes poor glioma prognoses, we applied the GSEA enrichment method. As shown in **Figure S6**, GNG12 was related to some signaling pathways that participate in the occurrence and development of cancer. Although GSEA only indirectly revealed the mechanism of GNG12 in promoting glioma, these results were based on a comparison of GNG12 with thousands of genes. Several researchers have used this method to identify promising biomarkers of gliomas; one such example was Xu and Liu (32, 33). Therefore, the results obtained from the present study were scientific and can be confirmed. Importantly, we conducted WB to further verify this result, which showed that targeted downregulation of GNG12 may impact key targets of the cell adhesion molecule pathway, including ICAM-1, VCAM-1, and CDH2 (34). Other studies have shown that cell adhesion molecules play an essential role in the malignant progression of tumors, and that downregulating key molecular targets can inhibit tumor proliferation and migration (35). Among them, ICAM-1, a cell surface glycoprotein and adhesion receptor, can easily influence inflammatory responses and strongly impacts tumor cell survival and propagation (36). Furthermore, VCAM1 derived from cancer-associated fibroblasts (CAFs) interacts with integrin αvβ1/5 in gastric cancer and promotes tumor invasion in the organism (37). This could prove that our study’s results represent a step in the right direction. In conclusion, this study has objectively verified that GNG12 contributes to glioma development and poor prognoses by regulating cell adhesion molecular pathways.

During this study, we conducted a scientific in-depth analysis using a large sample of data from multiple databases, and some unavoidable limitations occurred. First, because some of the samples in this study were obtained from multiple databases, multicenter studies inevitably have some drawbacks, such as a possible bias in sample collection and detection methods. Second, the small size of the health samples obtained from public databases compared with the sample size of the tumor tissues may lead to statistical errors. Finally, specific and personalized treatment information, such as the extent of surgical resection and tumor morphological characteristics, was unfortunately not available in the database based analysis. Therefore, we used *in vitro* cellular assays to verify that the knockdown of GNG12 significantly inhibited the proliferation and migration abilities of glioma cell lines and explored some of the molecular mechanisms of GNG12 in cell signaling pathways. We accordingly reduce the errors caused by incomplete information in the database and other uncontrollable factors.

## Conclusion

First, our study showed that GNG12 is overexpressed in gliomas, and that there are some common clinical characteristics and molecular staging that are closely related to GNG12 expression. A high expression level of GNG12 often predicts a poor prognosis in patients with gliomas. Reducing GNG12 expression levels may inhibit tumor cell proliferation and invasion. Furthermore, GNG12 may regulate glioma development and progression by participating in the cell adhesion molecule pathway. Finally, this study provides a new molecular biological target for improving the prognosis and prolonging the survival of glioma patients, and provides important basic support for attacking the pathogenesis of glioma.

## Data Availability Statement

The raw data supporting the conclusions of this article will be made available by the authors, upon reasonable request. Requests to access the datasets should be directed to lrz13633858566@163.com.

## Author Contributions

ZL and YG developed the original idea and the protocol, abstracted and analyzed data, RL and XC wrote the manuscript, and is the guarantor. All authors contributed to the article and approved the submitted version.

## Funding

This work was supported by The Thousand Talents Plan of Central Plains (ZYQR201912122).

## Conflict of Interest

The authors declare that the research was conducted in the absence of any commercial or financial relationships that could be construed as a potential conflict of interest.

## Publisher’s Note

All claims expressed in this article are solely those of the authors and do not necessarily represent those of their affiliated organizations, or those of the publisher, the editors and the reviewers. Any product that may be evaluated in this article, or claim that may be made by its manufacturer, is not guaranteed or endorsed by the publisher.

## Supplementary Material

Supplementary Table 1 Characteristics of patients with glioma based on CGGA. CGGA, Chinese Glioma Genome Atlas; WHO, World Health Organization; IDH, Isocitrate dehydrogenase.

Supplementary Table 2 Primer sequence of GNG12. siRNA sequences with the highest knockdown efficiency of GNG12.
