# Supplementary material for: GNG12 as A Novel Molecular Marker for the Diagnosis and Treatment of Glioma
Source: Front Oncol. 2022 Jul 19;12:726556. doi: 10.3389/fonc.2022.726556 (PMC9345608; doi:10.3389/fonc.2022.726556)
Supplement: Supplementary Table 2 — Primer sequence of GNG12. siRNA sequences with the highest knockdown efficiency of GNG12. [file Table_2.docx]

**TABLE S2**

**Primer sequence of GNG12**

| **Gene** | **Primer sequences (5’-3’)** |
| --- | --- |
| **GNG12-Forward** | **GAGCCCTTAGAGACCGAG** |
| **GNG12-Reverse** | **GACTTTGTGTGGTCCAATGT** |

**siRNA sequences with the highest knockdown efficiency of GNG12**

| **Gene** | **Sequence** |
| --- | --- |
| **siRNA-** **GNG12-Forward** | **GCGUGUGCCAGCCUGUAUATT** |
| **siRNA-** **GNG12-** **Reverse** | **UAUACAGGCUGGCACACGCTT** |
