# Supplementary figures and images for: GNG12 as A Novel Molecular Marker for the Diagnosis and Treatment of Glioma
Source: Front Oncol. 2022 Jul 19;12:726556. doi: 10.3389/fonc.2022.726556 (PMC9345608; doi:10.3389/fonc.2022.726556)

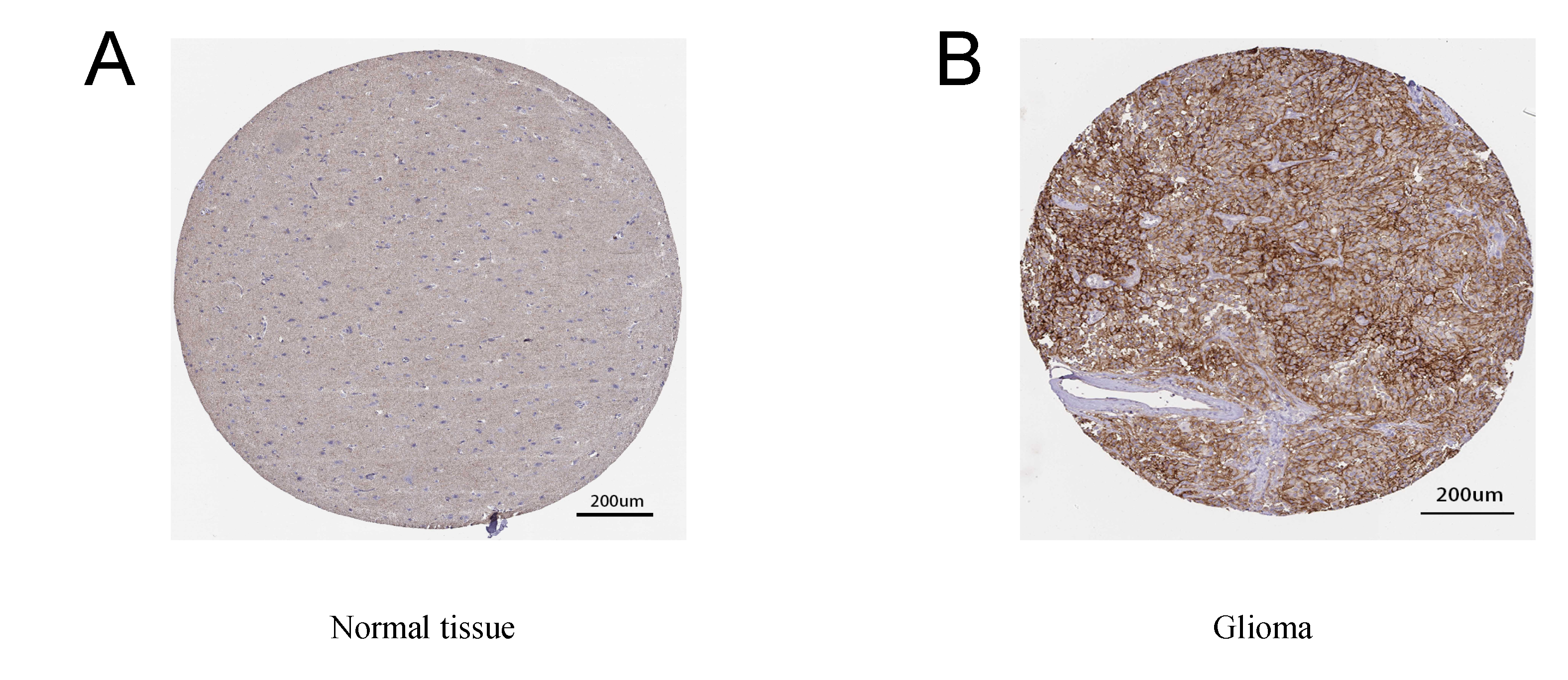

Supplement: Supplementary file 3 [file Image_1.tif]

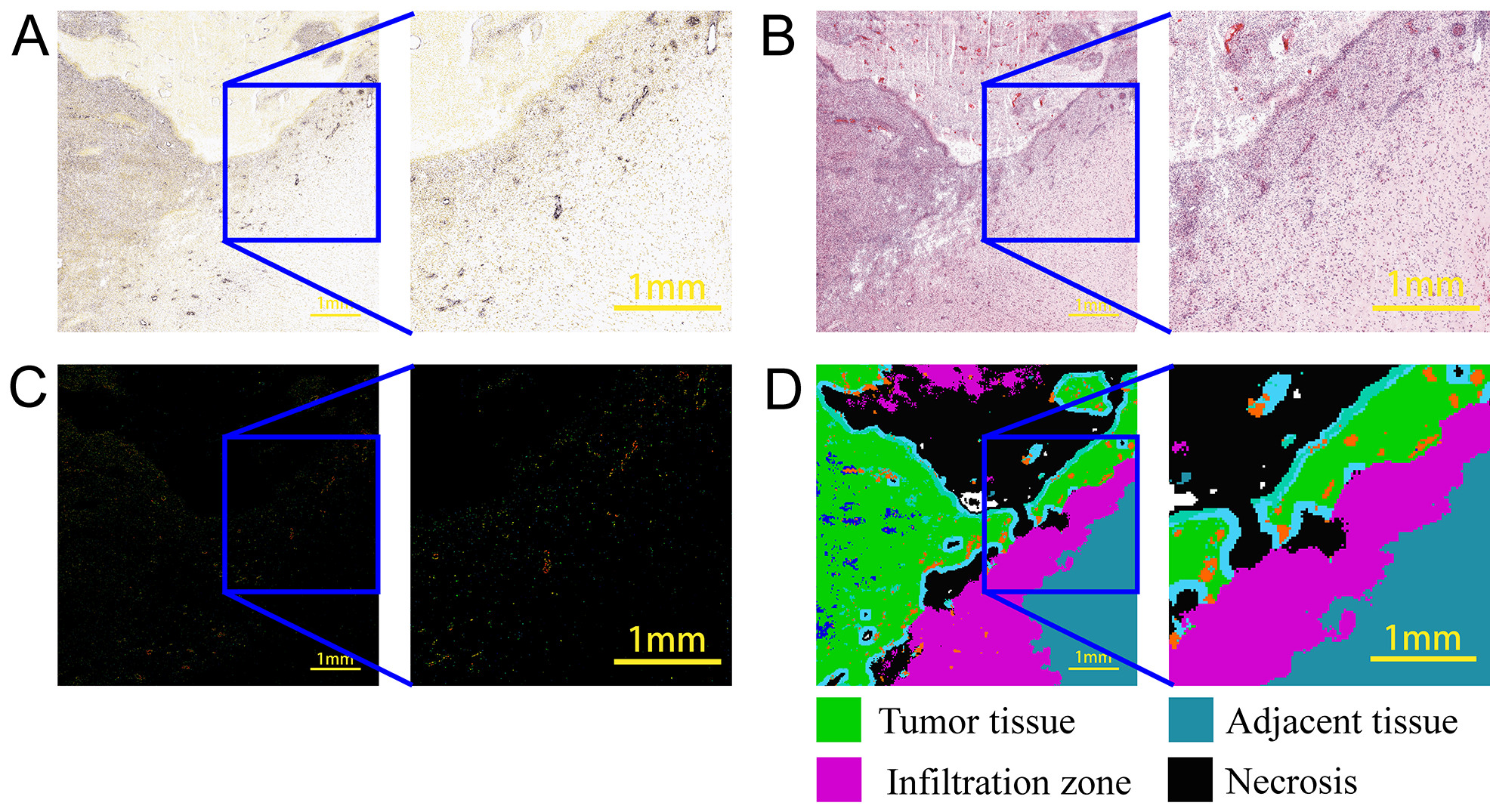

Supplement: Supplementary file 4 [file Image_2.tif]

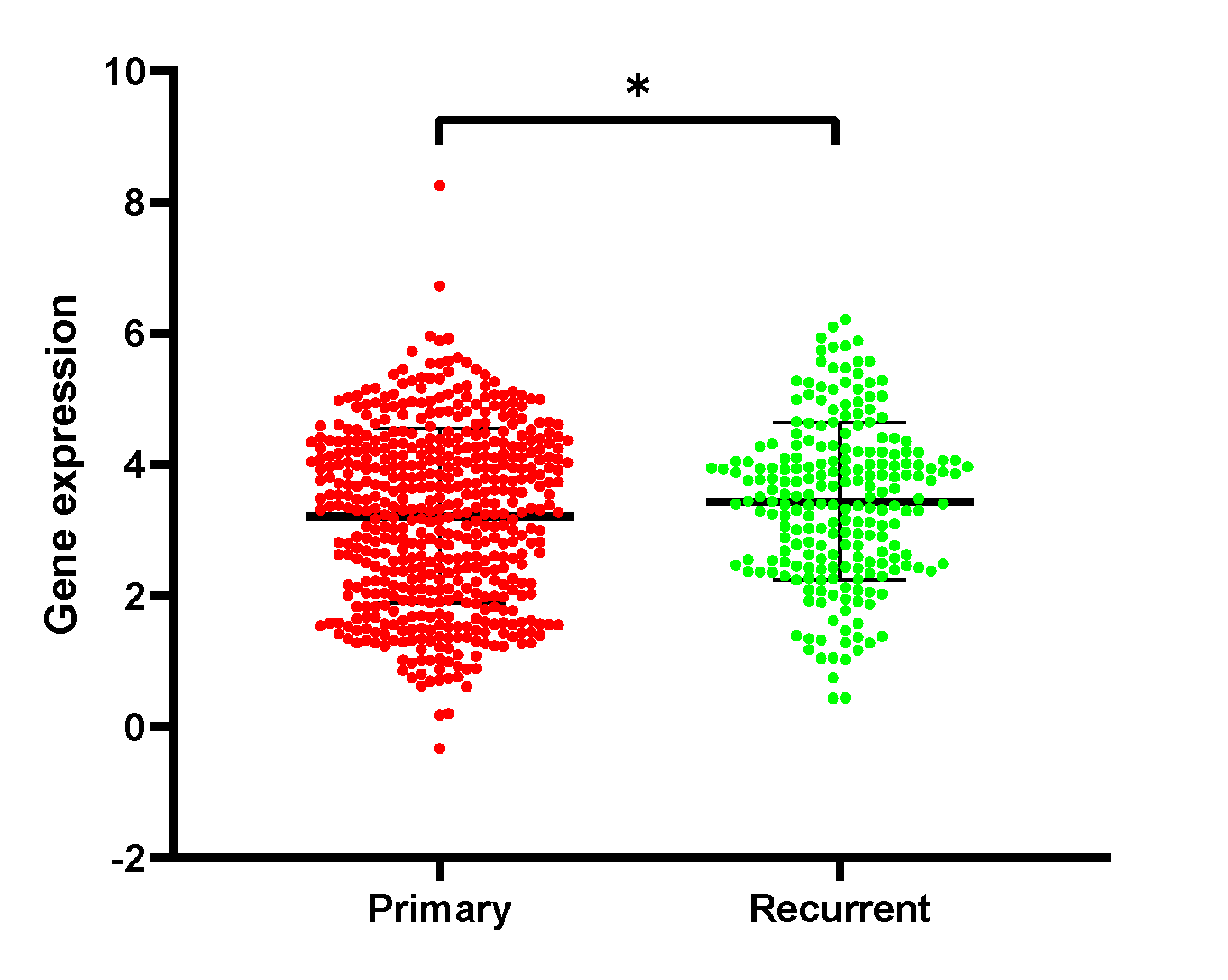

Supplement: Supplementary file 5 [file Image_3.tif]

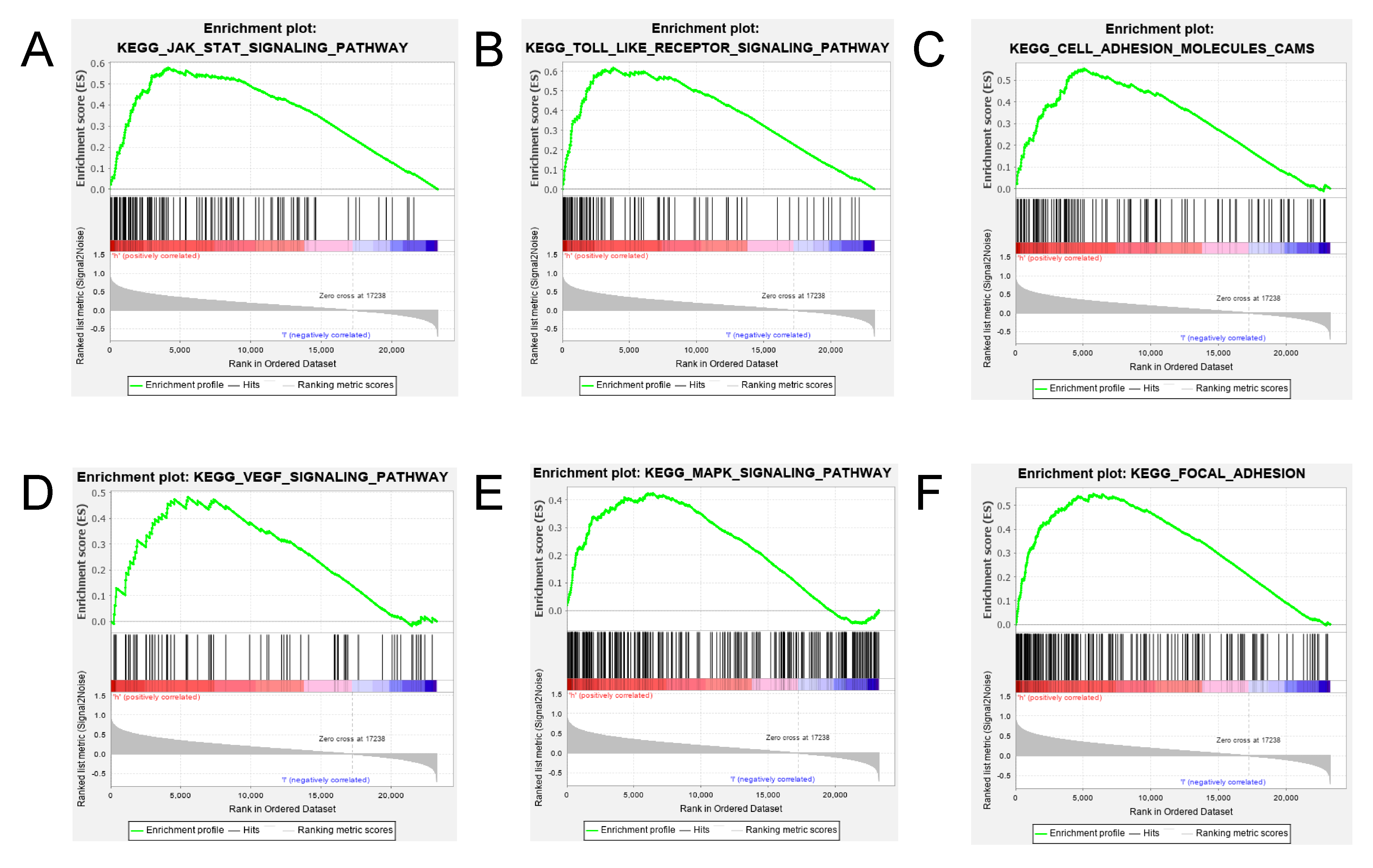

Supplement: Supplementary file 8 [file Image_6.tif]
